# Characterization of Two Endo-β-1, 4-Xylanases from *Myceliophthora thermophila* and Their Saccharification Efficiencies, Synergistic with Commercial Cellulase

**DOI:** 10.3389/fmicb.2018.00233

**Published:** 2018-02-14

**Authors:** Abdul Basit, Junquan Liu, Ting Miao, Fengzhen Zheng, Kashif Rahim, Huiqiang Lou, Wei Jiang

**Affiliations:** ^1^Beijing Advanced Innovation Center for Food Nutrition and Human Health, MOA Key Laboratory of Soil Microbiology, College of Biological Sciences, China Agricultural University, Beijing, China; ^2^Beijing Key Laboratory of Genetic Engineering Drug and Biotechnology, Institute of Biochemistry and Biotechnology, College of Life Sciences, Beijing Normal University, Beijing, China

**Keywords:** *Myceliophthora thermophila*, xylanases, *Pichia pastoris*, expression, saccharification

## Abstract

The xylanases with high specific activity and resistance to harsh conditions are of high practical value for biomass utilization. In the present study, two new GH11 xylanase genes, *MYCTH_56237* and *MYCTH_49824*, have been cloned from thermophilic fungus *Myceliophthora thermophila* and expressed in *Pichia pastoris*. The specific activities of purified xylanases reach approximately 1,533.7 and 1,412.5 U/mg, respectively. Based on multiple template-based homology modeling, the structures of their catalytic domains are predicted. Enzyme activity was more effective in 7.5 L fermentor, yielding 2,010.4 and 2,004.2 U/mL, respectively. Both enzymes exhibit optimal activity at 60°C with pH of 6.0 and 7.0, respectively. Their activities are not affected by EDTA and an array of metal ions. The kinetic constants have been determined for MYCTH_56237 (*K*_*m*_ = 8.80 mg/mL, *V*_*max*_ = 2,380 U/mg) and MYCTH_49824 (*K*_*m*_ = 5.67 mg/mL, *V*_*max*_ = 1,750 U/mg). More importantly, both xylanases significantly cooperate with the commercial cellulase Celluclast 1.5 L in terms of the saccharification efficiency. All these biochemical properties of the xylanases offer practical potential for future applications.

## Introduction

Plant biomass needs to be explored as a significant source of components that can be converted into products used in industrial processing. It is the most abundant renewable source of energy and mainly consists of lignocellulose, the complicated heterogeneous complex made up of hemicellulose, lignin and cellulose (Tuck et al., 2012). Recently, researchers have focused on developing different innovative technologies to convert lignocellulosic materials into useful industrial products (Thanaporn et al., 2015). The lignocellulosic materials and their efficient biodegradation are among the best areas of interest for researchers on account of their potential application in industrial activities (Govumoni et al., 2013). Among them, the major component of hemicellulose, xylan, has a high capability for conversion to applicable products.

Xylanases are the large group of hydrolytic enzymes that catalyze the hydrolysis of 1, 4-β-D-xylosidic linkages in the xylan backbone of hemicellulose. Xylanases play a vital role in the efficient saccharification of lignocellulose materials and are of great interest for their capability to hydrolyze biomass to fermentable sugars (Zhang et al., 2011). However, the high cost of commercial enzymes creates a bottleneck for their industrial applications. Various strategies have been applied to reduce the cost, such as improving enzyme activity, using crude extract enzymes or enzyme cocktails and maximizing enzyme production (Del Pozo et al., 2012; Lin et al., 2017).

The distinctive features of xylanases are based on their substrate specificities, modes of action, 3D structures and biochemical properties. The application of genetic engineering has helped the mass-scale production and identification of a wide variety of novel xylanases (Uday et al., 2016). The developments in modern recombinant protein technology have also paved the way for the expression of xylanases in both homologous and heterologous hosts. The recombinant strains are producing a high yield of xylanases for industrial application with less downstream purification. Thus xylanase production will be cheaper and cost efficient (Lu et al., 2016). *Pichia pastoris* is known for its properties of efficient enzyme secretion and fast growth with high cell density in simple media (Lu et al., 2016). Furthermore, unlike the bacterial expression system, *Pichia pastoris* plays a key role in the stability, proper folding and transportation of proteins (Ng et al., 2011). Moreover, *P. pastoris* is commonly used for recombinant protein production with the capability of adding both O- and N-linked carbohydrate moieties to the secreted proteins (Halim et al., 2015).

There is a growing interest in exploring the enzyme-producing thermophilic microorganisms toward the efficient saccharification of lignocellulosic materials for its biotechnological application. The xylanase stability at high temperature is of great concern for the efficient degradation of lignocellulose biomass because saccharification is usually performed at high temperatures (Myat and Ryu, 2016). Filamentous fungi are prolific producers of xylanolytic enzymes and (Andersen et al., 2016). A thermophilic fungus, *Myceliophthora thermophila*, is considered one of the most powerful cellulolytic organisms that synthesize the necessary cell wall–degrading enzymes. It is used for industrial enzyme production in new advanced technologies, such as the biomass-derived fuels. It has a comparatively high number of arabinoxylan-degrading enzymes and lignocellulolytic enzymes and it synthesizes a complete set of enzymes essential for cellulose degradation (Berka et al., 2011). Sequence analysis of the *M. thermophila* genome has revealed an extensive repertoire of genes responsible for the production of thermostable enzymes such as proteases, carbohydrate-active enzymes, oxidoreductases, lipases, and xylanases (Berka et al., 2011). Such potency of *M. thermophila* in association with its published complete genome sequence raised our interest for exploring xylanases from this fungus.

The present study describes the cloning and expression of two novel xylanase-encoding genes from *M. thermophila* belonging to family GH11, with their heterologous expression using the methylotrophic yeast *P. pastoris* expression system. Furthermore, the enzyme properties are characterized and their saccharification efficiency in combination with a commercial cellulase is examined. Although endo-xylanases from other fungi have been previously characterized (Ustinov et al., 2008; Fang et al., 2014; Lu et al., 2016), the properties of the recombinant xylanases from *M. thermophila* have some characteristic features regarding catalytic efficiency and enzyme properties.

## Materials and methods

### Strains, plasmids, reagents, and media

*Myceliophthora thermophila* ATCC 42464 was used as the source of genomic DNA (purchased from ATCC). *P. pastoris* (strain X-33), *E. coli* (strain DH5α) and expression vectors pPICZαA are in storage in our laboratory. Yeast extract peptone dextrose (YPD) and buffered minimal glycerol (BMGY) media were used for the cultivation of *P. pastoris*. Gene expression was induced in buffered minimal methanol medium (BMMY). All the above mentioned mediums were prepared according to the manual in the *Pichia* Expression Kit (Invitrogen). The T simple vector (code D104A), restriction enzymes and ligases were purchased from Takara Biotechnology (Dalian, China). PCR reagents, DNA markers and purification kits were purchased from Beijing HT Biotech Co. Ltd. Protein markers were from Thermo Scientific. Remazol brilliant blue-xylan (RBB-xylan) used for measuring xylanase activity was purchased from Sigma Aldrich (M5019). Other chemicals are analytical grade reagents unless otherwise stated.

### Construction of the recombinant plasmids

According to the reported genome sequence of *M. thermophila* (ATCC 42464) from the NCBI (National Center for Biotechnology Information) database, two xylanase genes, *MYCTH_56237* (Gene ID: 11506578, 672 bp) and *MYCTH_49824* (Gene ID: 11509563, 693 bp), were selected for cloning. The two gene sequences of *MYCTH_56237* and *MYCTH_49824* (without the signal peptide coding sequence based on SignalP 4.0 prediction) were optimized using the JAVA Codon Adaptation Tool (JCAT) (http://www.jcat.de/Start.jsp), removing rare codons and optimizing the codon usage for expression. The full-length xylanase gene fragments inserted into the pMD18-T simple vector, denominated as pMD18-T-MYCTH_56237 and pMD18-T-MYCTH_49824, respectively, were constructed by Tsingke Biotech. The two xylanase genes were amplified by PCR-specific primers, designed for expression in *P. pastoris* (primers are listed in Supplementary Table with the enzymatic restriction sites). All PCR products were amplified under the following conditions: initial denaturation at 95°C for 5 min; 35 cycles of denaturation at 95°C for 30 s, annealing at 58°C for 30 s and polymerization at 72°C for 1 min 30 s; and a final extension at 72°C for 10 min.

### Gene cloning and sequence analysis

The resulting PCR product was purified from the gel [Tiangen, Biotech (Beijing) Co. Ltd., China]. The gel-purified PCR products of the two xylanase gene fragments (*MYCTH_56237* and *MYCTH_49824*) were inserted into endonuclease restriction sites of *EcoR*I and *Xba*I of the recombinant plasmid pPICZαA and were transformed in the *E. coli* strain DH5α. The positive transformants were selected on low-salt LB plates and were further confirmed by PCR using specific primers (Supplementary Table) and DNA sequencing (Tsingke Biotech). The successful recombinant strains were then cultured overnight and the two plasmids were extracted [Tiangen, Biotech (Beijing) Co. Ltd., China]. The *Sac*I linearized pPICZαA plasmids were transformed into *P. pastoris* strain X-33 by electroporation according to the protocol given by the *Pichia* expression manual. The transformed cells were further screened on YPD agar plates containing Zeocin (100 μ g/mL). The integration of the target genes into the *P. pastoris* genome was confirmed by PCR using both specific and alcohol oxidase (AOX) primers (Supplementary Table). A vector-only control strain was prepared by transforming *P. pastoris* strain with the empty vector pPICZαA.

Nucleotide and protein sequences of both genes were aligned using the BLAST programs (http://www.ncbi.nlm.nih.gov/BLAST/). Vector NTI Advance 10.0 and DNAMAN 6.0 software were used to analyze the sequences. Signal peptides and glycosylation sites were predicted by the SignalP 4.1 server (http://www.cbs.dtu.dk/services/SignalP/) and the NetNGlyc 1.0 Server (http://www.cbs.dtu.dk/services/NetNGlyc/). Molecular weight and *pI* of deduced proteins were predicted using the ProtParam tool of ExPASY (http://web.expasy.org/). ClustalW software was used to perform the multiple sequence alignment and phylogenetic evaluation used the neighbor joining method with a Poisson model, conducted with MEGA software version 7.0. The reliability of the branching order was determined by 1,000 bootstrap replications.

### Expression of recombinant xylanases and purification

The recombinant colonies of *P. pastoris* were cultured in 5 mL tubes of BMGY medium at 30°C, 200 rpm for 12 h until the OD_600_ of the culture reached 4–6. The culture was then resuspended in 50 mL of BMMY containing 1.34% YNB and biotin (4.0 μg/mL) in a 250 mL shake flask. Pure methanol was added to the culture to a final concentration of 1% every 24 h to maintain induction. All induction experiments were performed in triplicate.

The expressed xylanases in *P. pastoris* were purified after removal of cell debris by centrifugation for 15 min at 12,000 rpm. The supernatant (containing the xylanase labeled with histidine tag) was adjusted to 10 mM imidazole and loaded onto a 1 mL HisTrap Chelating Ni-affinity column (Bio-Beads™, Sweden) equilibrated with 1× phosphate buffer (PB, 10 mM imidazole). The adsorbed proteins were eluted using a linear gradient of imidazole (50–500 mM) and both recombinant enzymes were eluted with 100 mM imidazole.

### SDS-PAGE, western blotting and deglycosylation analysis

The purified culture supernatants were pooled and assayed by 12% sodium dodecyl sulfate-polyacrylamide gel electrophoresis (SDS-PAGE). Protein bands were visualized by staining with Coomassie brilliant blue dye R-250 (Bio-Rad) or transferred to PVDF membrane for Western blotting. The membrane was incubated with blocking buffer, then with the c-Myc antibody (clone 9E10, Sigma) and the alkaline phosphatase-conjugated anti-mouse IgG (Jackson). The products were visualized by BCIP/NBT method. Protein bands with the predicted sizes on the SDS-PAGE were recovered and their concentrations were determined through Bradford method by using bovine serum albumin as standard (Bio-Rad protein assay kit, Bio-Rad Laboratories, Inc.).

Furthermore, the expressed MYCTH_56237 and MYCTH_49824 were deglycosylated by an endo-H enzyme (according to the protocol provided by New England Biolabs). Briefly, 1 mg of purified xylanase was dissolved in citrate buffer (pH 4.8) and denatured by boiling for 10 min. Then, it was treated with 0.1 U endo-H enzyme and incubated at 37°C for 1 h. Finally, the product was analyzed on SDS-PAGE (12%).

### Enzyme activity assay

Enzyme activity of the xylanases was assayed using RBB-xylan as the substrate, as described by Yin et al. (2010). The standard assay mixtures (100 μL) consisted of 25 μL purified enzyme, 25 μL sodium acetate buffer (100 mM, pH 5) and 50 μL 1.15% RBB-xylan. The reaction mixture was then incubated for 10 min at 60°C. The reaction was stopped by the addition of 200 μL ethanol (100%), which precipitates the residual substrate. The mixture was then incubated at room temperature for 15–20 min. The OD of the supernatant from the precipitated residual substrate collected by centrifugation (12,000 rpm for 5 min) was measured at 595 nm, in correspondence to the respective substrate blanks (Ahsan et al., 2001; Zhengqiang et al., 2001). One unit of xylanase activity was defined as the amount of enzyme that liberated RBB dye from the substrate, causing a 1 OD increase in the reaction mixture per minute under the assay conditions described above (Zhengqiang et al., 2001; Yin et al., 2010). All determinations were performed in triplicate for statistical analysis or the calculations of specific activity.

### Biochemical characterization of xylanases

To investigate the biochemical properties of purified recombinant MYCTH_56237 and MYCTH_49824, RBB-xylan was used as the substrate for the activity assay. For determinations of optimum pH, several buffers of 50 mM were used as follows: HCl-glycine (pH 2-3), C_6_H_8_O_7_-C_6_H_5_${\text{O}}_{7}^{3-}$ (pH 3-4), CH_3_COOH-CH_3_COO^−^ (pH 4-6), H_2_${\text{PO}}_{4}^{-}$- ${\text{HPO}}_{4}^{2-}$ (pH 6-8), Tris-HCl (pH 8-9). The pH stability of MYCTH_56237 and MYCTH_49824 was estimated by pre-incubating the enzymes in the above-mentioned buffers without substrate at 37°C for 60 min. The residual activities of the enzymes were evaluated at the optimum pH (MYCTH_56237: pH 6.0, MYCTH_49824: pH 7.0) under the same conditions mentioned above.

The optimal temperature of the purified enzymes was determined over the temperature range 40–80°C in 10°C steps. The relative enzyme activity at the optimum pH (MYCTH_56237: pH 6.0, MYCTH_49824: pH 7.0) was measured. Similarly, the effect of temperature on enzyme stability was investigated by determining the residual enzyme activity under standard conditions after preincubation of the purified enzyme at 60, 70, and 80°C in optimal pH without substrate for 30 min.

The xylanase activity of purified MYCTH_56237 and MYCTH_49824 was measured in the presence of 1 mM concentration of various metal ions (Mg^2+^, Ca^2+^, Co^2+^, Fe^2+^, Mn^2+^, Ni^2+^, Zn^2+^, Cu^2+^) and different chemical reagents, including SDS (0.1%), EDTA (1 mM) and Tween-20 (0.05%), under the standard reaction conditions for the estimation of their effects on xylanase activity. The control was used without any additive in the reaction mixture.

The kinetic parameters of MYCTH_56237 and MYCTH_49824 were investigated at different concentrations of substrate ranging from 1 to 12.5 mg/mL in 50 mM sodium acetate, pH 6.0 and 7.0, respectively, to determine the xylanase activity under standard conditions. The K_m_ and V_max_ values were evaluated by fitting the initial velocity data to a Michaelis-Menten kinetic model.

### Enzyme activity assay of commercial xylanases

RBB-xylan was used as the substrate for the activity assay of commercial xylanases. The xylanase activity of commercial xylanase(s), i.e., xylanase from *Trichoderma longibrachiatum* (X2629, Sigma-Aldrich), xylanase from *Thermomyces lanuginosus* (X2753, Sigma-Aldrich) and xylanase (Solarbio Life Sciences, X8091), was determined. The conditions were used as described above (optimum pH and temperature of each enzyme were sustained according to the information provided). Finally, the enzyme activities of MYCTH_56237 and MYCTH_49824 were compared with those of the above-mentioned commercial xylanases.

### Expression of recombinant xylanases in a bioreactor

Expression of the recombinant xylanases was carried out in a 7.5 L fermentor (Shanghai Boxing Bio-engineering Equipment Co. Ltd.) with 4 L BSM supplement containing 17.3 mL PTM1. Fermentation of MYCTH_56237 and MYCTH_49824 was applied based on the *Pichia* Fermentation Process Guidelines (Invitrogen). The temperature and pH were adjusted at 30°C and 5.0, respectively. In the cell growth phase, yeast cells were allowed to grow until the complete utilization of glycerol, which was indicated by the increase of dissolved oxygen (DO) level. When the glycerol was fully utilized from the feeding medium containing 50% (w/v) glycerol, 12 mL/L PTM1 solution was fed to fermenter according to a pre-adjusted DO level (10%). When the DO level reached higher than 60%, the glycerol feeding was stopped and pure methanol containing 12 mL/L PTM1 solution was pumped to induce the targeted gene expression, with the DO being set to 10–20%. Culture samples were taken every 8 h to determine the OD_600_ and enzyme activity.

### Saccharification of the pretreated corn stover

The pretreated corn stover (by 1% NaOH at 121°C for 30 min, washed with ddH_2_O and dried) was mixed with 20 mL of Na_2_HPO_4_-Citric acid buffer (100 mM, pH 5.0) in 50 mL flasks at 2% concentration (dry material). Celluclas 1.5 L (cellulase from *Trichoderma reesei* ATCC 26921, C2730, Sigma-Aldrich) was supplemented with the purified recombinant xylanases in the cellulosic preparations. The reaction mixture was incubated at 50°C at 200 rpm for 72 h. After several intervals, the collected hydrolyzates were centrifuged at 12,000 rpm for 10 min. The amounts of reducing sugars released in the supernatants were determined using the DNS method. The experiment was performed in triplicate.

The amount of reducing sugar was determined by DNS colorimetry with glucose as the standard (Miller, 1959). The calculation of the corn stover degradation (%) was calculated using the following equation:

(1) $$\begin{array}{r} \text{Degradation of corn stover }\left(\%\right)\;=\;\left(\frac{\text{The reducing sugars obtained by enzymatic hydrolysis }\left(\text{mg}\right)}{\text{Amount of pretreated corn stover used }\left(\text{mg}\right)}\right)\times 100\% \end{array}$$

### Homology modeling

The secondary structure (2D) predictions for MYCTH_56237 and MYCTH_49824 were performed in the ESPript 3 (at http://espript.ibcp.fr/ESPript/ESPript/). The 3D structural models were accomplished by the SWISS-MODEL (http://www.expasy.org/swissmod/) and PyMol Molecular Graphics System, was used to visualize and generate the graphical figures of the constructed models. The appropriate template was decided for specific protein modeling based on the score interpreted by HHpred. Moreover, the quality of the constructed tertiary structure of the protein model was assessed by the ProQ server (http://proq.bioinfo.se/ProQ/ProQ.html) (Wallner and Elofsson, 2003).

## Results

### Sequence analysis

Both *MYCTH_56237* (672 bp) and *MYCTH_49824* (693 bp) display sequence identity with endo-β-1,4-xylanases belonging to GH11 family glycoside hydrolase according to the information available on the NCBI and CAZy database (http://www.cazy.org/) GH11. *MYCTH_56237* and *MYCTH_49824* encode proteins of 223 and 230 amino acids, with the predicted masses of 23,776.90 and 25,338.72 Da, respectively. MYCTH_56237 and MYCTH_49824 were predicted by SignalP to harbor the signal peptides for secretion and (Supplementary Figure 1). According to the ProtParam tool of ExPASY, the theoretical isoelectric points (pI) are 5.0 and 6.18, respectively. To enhance the probability of expression level, the genes *MYCTH_56237* and *MYCTH_49824* were optimized by upgrading the codon adaptation index (CAI) from 0.093 to 0.97 and 0.08 to 0.97, respectively. After codon optimization, the new sequence of the *MYCTH_56237* was changed by 25.6% from the native gene and the GC content was reduced from 66.8% to 44.3%. Similarly, the gene sequence of *MYCTH_49824* was changed by 27.3% and the GC content was reduced from 64.6 to 43.7%.

### Phylogenetic analysis

**Figure 1:**
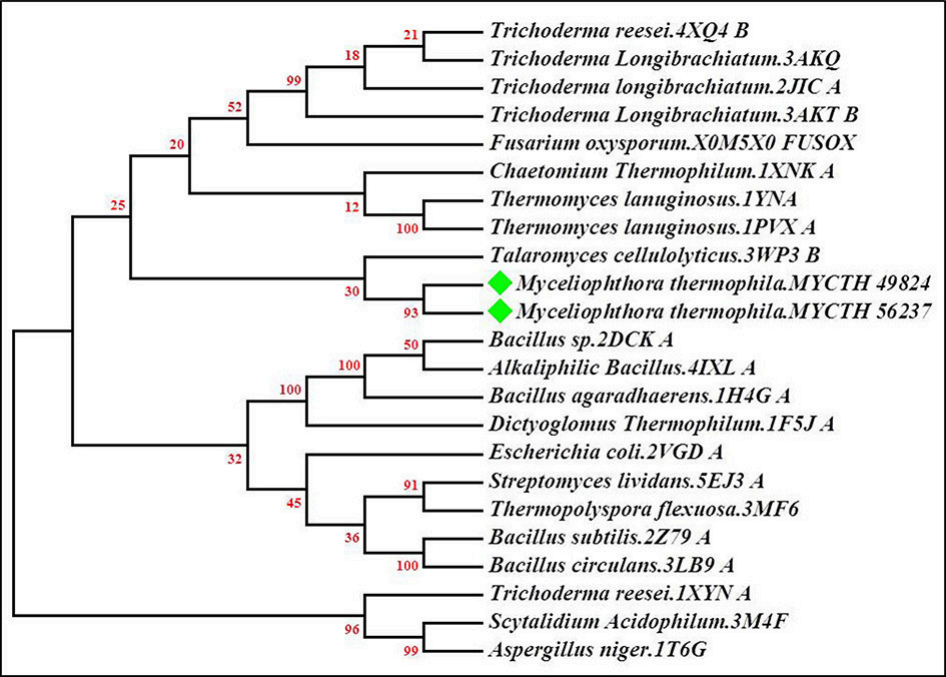
Phylogenetic tree of xylanases (*MYCTH_56237* and *MYCTH_49824*). The tree of selected xylanase sequences was generated by using the neighbor-joining (NJ) method with Poisson model as implemented in Mega software 7.0. *MYCTH_56237* and *MYCTH_49824* are labeled with green diamonds. PDB numbers of each xylanase are given after each species name. The lengths of the branches show the relative divergence among amino acid sequences of the xylanases and the scale bar indicates the amino acid substitutions per position. Bootstrap values are shown at nodes.

A phylogenetic tree of *M. thermophila-*derived endo-xylanases (MYCTH_56237 and MYCTH_49824) was constructed based on the sequence similarity of the amino acids with its closest homologs derived from the HHpred analysis tool (https://toolkit.tuebingen.mpg.de/hhpred). A total of 20 hit sequences out of 31 were then aligned through ClustalW using neighbor-joining algorithm method and Poisson model with the booststrap stimulation in MEGA 7.0 (Figure 1). The sequence alignment and phylogenetic analyses of MYCTH_56237 and MYCTH_49824 showed the highest identity (57–63%) with the xylanases from *Talaromyces cellulolyticus* (3Wp3), *Trichoderma longibrachiatum* (3akq_A), *Chaetomium thermophilum* (1xnk_A) and *Thermomyces lanuginosus* (1yna_A). Moreover, the two xylanases showed 50–55% identity with the xylanases from *E. coli* (2vgd_A), *Streptomyces lividans* (5ej3_A), *Thermopolyspora flexuosa* (3mf6_A), *Bacillus subtilis* (2z79_A), *Dictyoglomus thermophilum* (1f5j_A), *Bacillus subtilis* (1axk_A), and *Bacillus circulans* (3lb9_A).

### Expression of recombinant xylanases

For the expression in *P. pastoris*, the expression plasmids harboring the xylanase-encoding genes (*MYCTH_56237* and *MYCTH_49824*) were cloned in fusion with the signal sequence of the α-factor in the *P. pastoris* vector (pPICZαA) and verified by DNA sequencing. Positive transformants were selected on YPD agar plates containing Zeocin (100 μg/mL). The transformants carrying the *MYCTH_56237* and *MYCTH_49824* genes were further under control of an AOX1 promoter, which is inducible by 1% (v/v) methanol. After 96 h of cultivation in BMMY medium, the xylanase activity of MYCTH_56237 and MYCTH_49824 in the culture supernatant was measured to be about 72.96 and 63.21 U/mL, respectively. The activity of MYCTH_56237 and MYCTH_49824 increased remarkably by one-step affinity purification on immobilized metal ion affinity chromatography (IMAC), at 1,533.71 and 1,412.46 U/mg, respectively.

**Figure 2:**
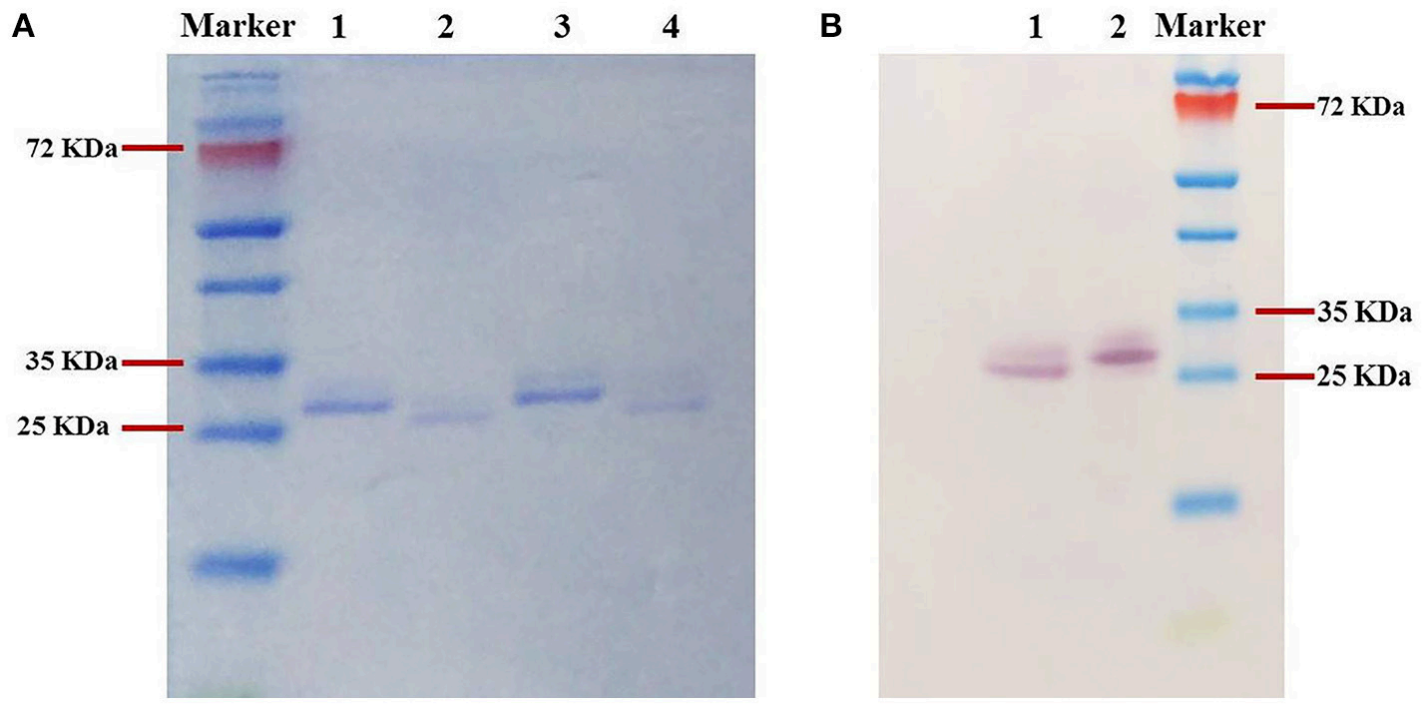
SDS-PAGE analysis **(A)** and Western blot analysis **(B)** of purified recombinant xylanases. **(A)** Lanes 1 and 3: Purified MYCTH_56237 and MYCTH_49824; Lanes 2 and 4: Deglycosylated MYCTH_56237 and MYCTH_49824 using endo H enzyme. **(B)** Lanes 1 and 2, MYCTH_56237 and MYCTH_49824, respectively.

Furthermore, in the SDS-PAGE analyses, the purified MYCTH_56237 and MYCTH_49824 migrated close to their theoretical molecular weights (Figure 2A, Lanes 1 and 3). The positive bands of both enzymes shown by western blotting were located at the same positions as the single bands on SDS-PAGE (Figure 2B, Lanes 1 and 2).

### Deglycosylation analysis

To estimate the effect of glycosylation on the activity of MYCTH_56237 and MYCTH_49824, the specific activity of each xylanase was determined following treatment with the endoglycosidase-H enzyme. Postdeglycosylation, MYCTH_56237 and MYCTH_49824 migrated slightly faster in SDS-PAGE (Figure 2A, Lanes 2 and 3), suggesting that they very likely contain glycosylation as expressed in *P. pastoris*. Indeed, MYCTH_56237 has two putative O-glycosylation sites (residues 35 and 37) and one N-glycosylation site (Asn22), while MYCTH_49824 has one O-glycosylation site (at residue 40) and four N- (resides 20, 33, 101, 107). Interestingly, the deglycosylated MYCTH_56237 showed 40% reduced xylanase activity compared to untreated one, whereas only subtle change was observed in MYCTH_49824. This might reflect the different sensitivity of these two enzymes to glycosylation.

### Characterization of recombinant xylanases

**Figure 3:**
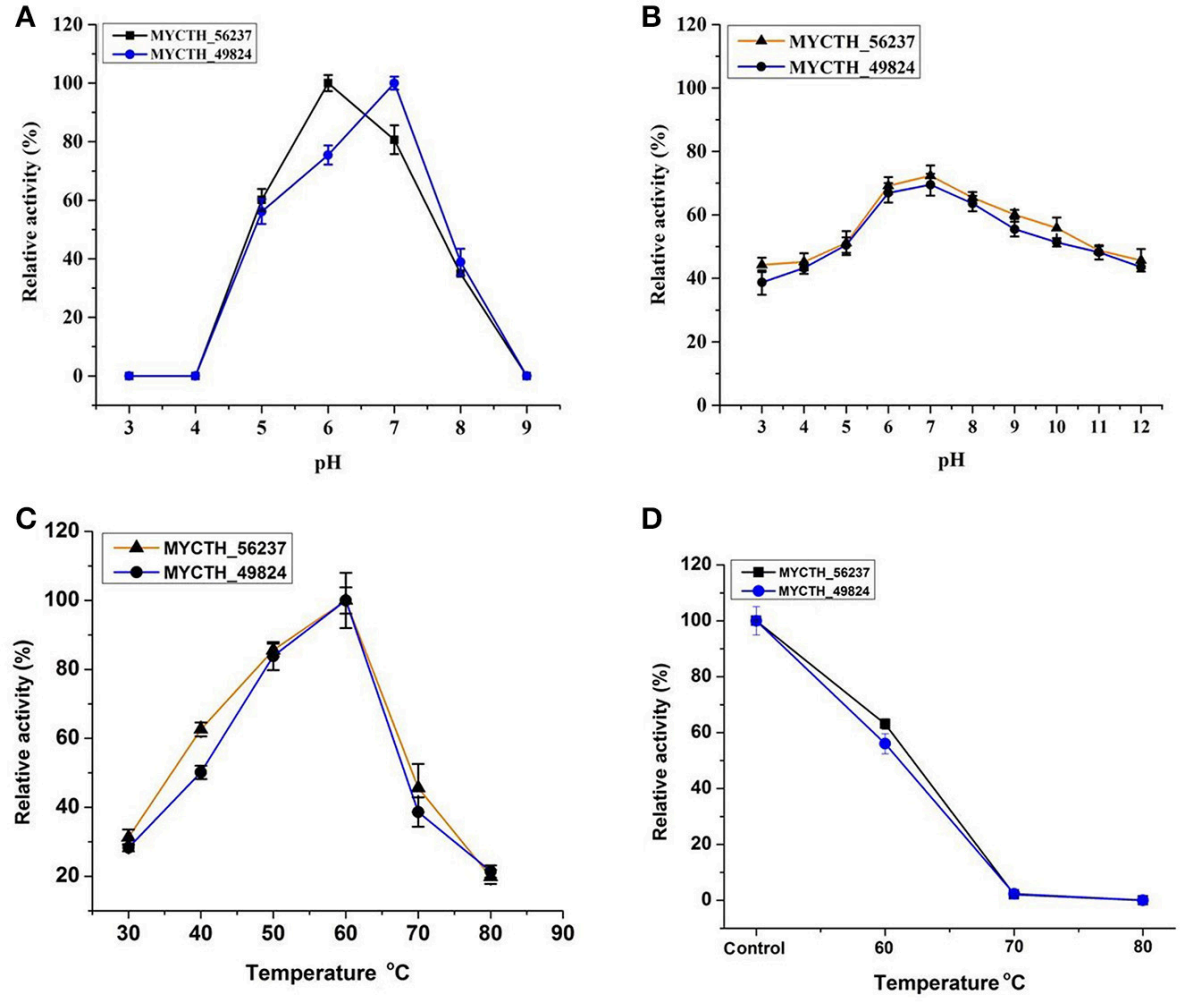
Enzymatic properties of the purified recombinant MYCTH_56237 and MYCTH_49824 in *P. pastoris* using RBB-xylan as the substrate. **(A)** Effect of pH on enzyme activity. **(B)** pH stability of MYCTH_56237 and MYCTH_49824. **(C)** Effect of temperature on enzyme activity. **(D)** Thermostability of MYCTH_56237 and MYCTH_49824 at their optimum pH.

Next, the properties of recombinant purified xylanases MYCTH_56237 and MYCTH_49824 expressed in *P. pastoris* were determined using RBB-xylan as the substrate. MYCTH_56237 and MYCTH_49824 were active over a wide range of pH (2.0 to 9.0), exhibiting optimal activity around pH 6.0 and 7.0, respectively (Figure 3A). After incubation for 60 min at the pH range of 2.0–12.0 at room temperature, MYCTH_56237 and MYCTH_49824 retained nearly 70% of the maximal activity at their optimal pH conditions (Figure 3B). Both MYCTH_56237 and MYCTH_49824 exhibited the highest activity at 60°C (Figure 3C). When the enzymes were pre-incubated at 70°C for 30 min, MYCTH_56237 and MYCTH_49824 retained about 60% activity at 60°C compared to the untreated one (Figure 3D). These results allow us to conclude that MYCTH_56237 and MYCTH_49824 have relatively better performance at 60°C, over a wide range of pH values compared to other GH11 family xylanases.

Then, the effects on xylanase activity of different metal ions and chemical reagents were determined. The enzyme activities of both xylanases were enhanced significantly in the presence of SDS and Tween-20. Mg^2+^ and Mn^2+^ enhanced the activity of MYCTH_56237 and MYCTH_49824 by more than 100% (Supplementary Figure 2). Meanwhile, the enzymes maintained an activity of more than 80% in the presence of Mg^2+^, Co^2+^, Ca^2+^, Mn^2+^, Fe^2+^ and EDTA, respectively. Moreover Cu^2+^, Zn^2+^, and Ni^2+^ maintained the activity of MYCTH_56237 and MYCTH_49824 by >60%, except Cu^2+^ in case of MYCTH_49824 (Supplementary Figure 2).

Kinetics parameters of MYCTH_56237 and MYCTH_49824 were evaluated for the hydrolysis of the RBB-xylan substrate at concentrations ranging from 1.125 to 18 mg/mL. MYCTH_56237 exhibited the *V*_*max*_ and *K*_*m*_ of 2,380 U/mg and 8.80 mg/mL, respectively, whereas MYCTH_49824 exhibited *V*_*max*_ and *K*_*m*_ of 1,750 U/mg and 5.67 mg/mL, respectively.

### Enzyme activity assay of commercial xylanases

The xylanase activities of MYCTH_56237 and MYCTH_49824 were also compared to that of commercial xylanases. The activities of commercial xylanases from *Trichoderma longibrachiatum* (Sigma) and *Thermomyces langinosus* (Sigma) toward RBB-xylan were determined to be 18.96 and 22.4 U/mg, respectively. The specific xylanase activities of MYCTH_56237 and MYCTH_49824 were 70-fold higher compared to the resulting specific activity of commercial xylanases.

### Expression of recombinant xylanases in a bioreactor

**Figure 4:**
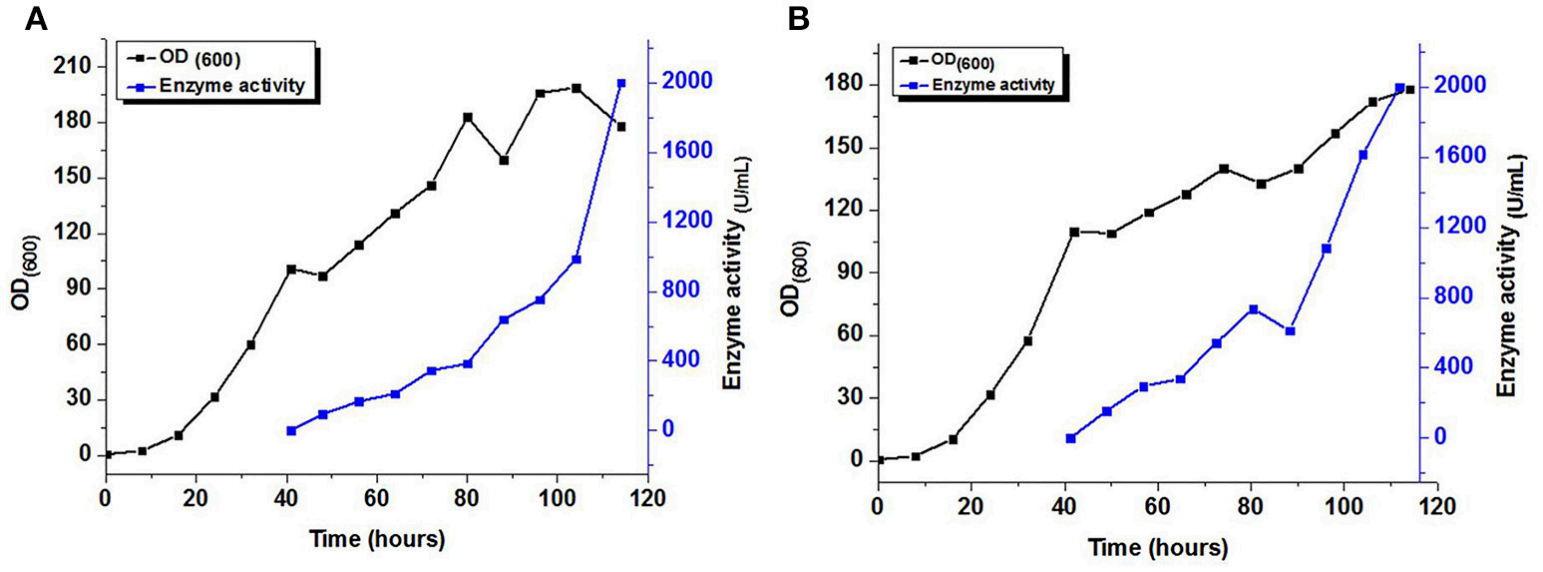
Expression of recombinant MYCTH_56237 **(A)** and MYCTH_49824 **(B)** in a 7.5 L fermentor for xylanase production.

Fed-batch fermentation with the MYCTH_56237 and MYCTH_49824 was carried out in a 7.5 L fermentor. The recombinant strain was inoculated and glycerol was employed after 24 h. Upon termination of the glycerol feed, the rate of DO was increased to above 50%. Methanol feeding was then employed to induce the production of xylanase with a stepwise increasing rate. No xylanase activity was detected until methanol feeding was initiated. After adding the methanol for 72 h, the maximum xylanase activity in the supernatant was 2,010.4 and 2,004.2 U/mL, respectively (Figure 4). These results for enzyme activities of MYCTH_56237 and MYCTH_49824 were much higher than those in the shake flask, i.e., 72.96 and 63.21 U/mL, respectively. This result indicates that the production of these enzymes could be further elevated through fermentation engineering.

### Saccharification of the pretreated corn stover

**Figure 5:**
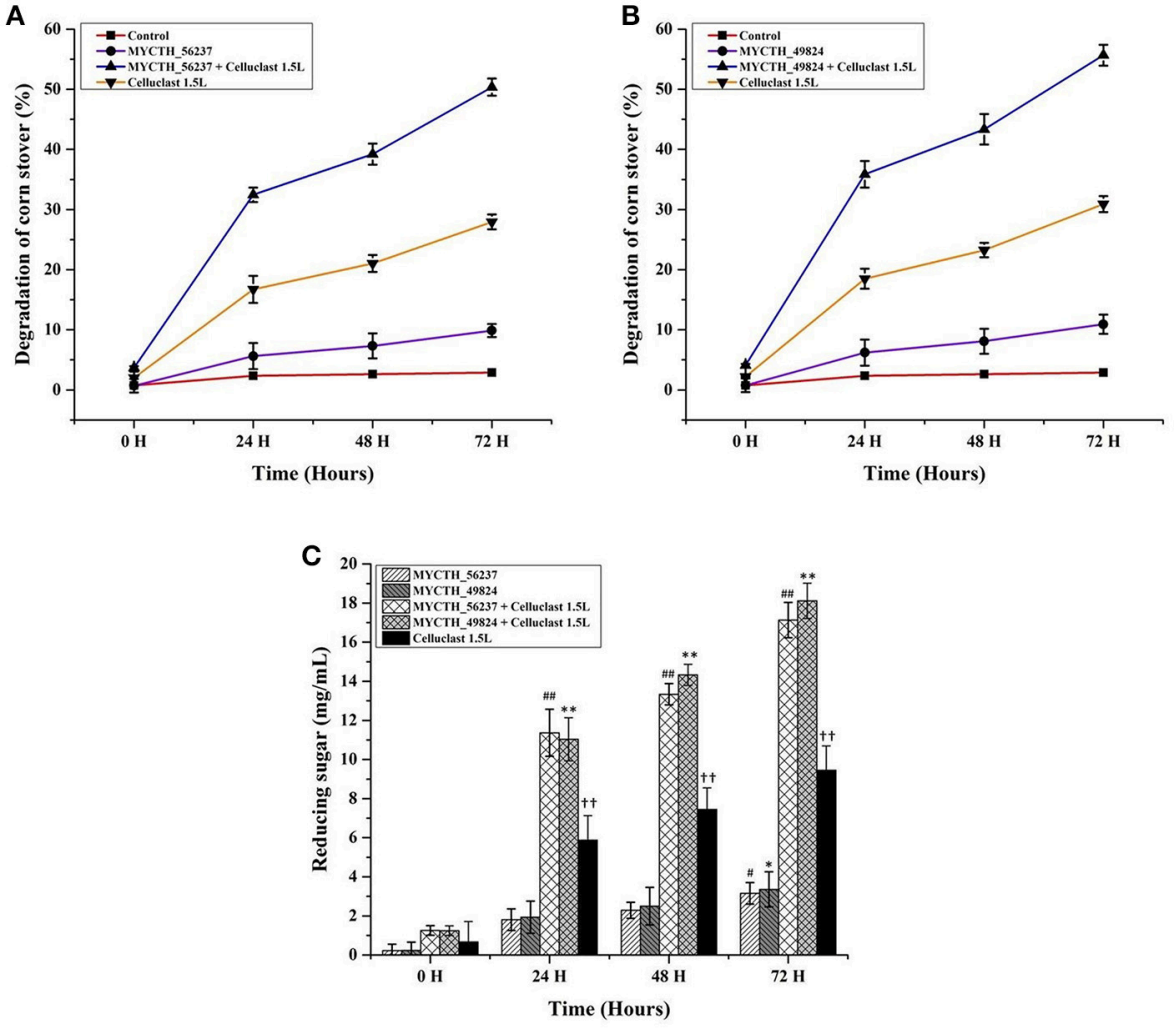
Saccharification efficiencies of MYCTH_56237 and MYCTH_49824 in combination with commercial Celluclast 1.5L (Sigma). The pretreated corn stover was used as substrate. **(A**,**B)** Represent the degradation (%) of corn stover in the combination with MYCTH_56237 (0.6 mg/g biomass) and MYCTH_49824 (1.06 mg/g biomass), respectively, in combination with commercial Celluclast 1.5L (1.38 FPU/g biomass). **(C)** Represents the reducing sugar released by the enzyme(s). Data represent mean ± *SD* of the independent tripilcates. **, ^##^,^††^ indicates *p* < 0.01; while ^#^, * indicates *p* < 0.05.

The commercial cellulase Celluclast 1.5 L (Sigma-Aldrich) from the mutant strain *T. reesei* (ATCC 26921) is the most commonly used in biomass degradation. Therefore, the application potential of MYCTH_56237 and MYCTH_49824 was evaluated by the saccharification of cellulosic materials in corn stover in combination with the commercial cellulase Celluclast 1.5 L. In the blank control group, Celluclast 1.5 L (1.38 FPU/g biomass) degraded about 30% of the corn stover over 72 h incubation at pH 5.0 and 50°C. When MYCTH_56237 and MYCTH_49824 were added at the dosage of 0.65 mg/g biomass and 1.06 mg/g biomass, respectively, the saccharification efficiencies were significantly improved to nearly 50.3 and 55.6% degradation of the corn stover (Figures 5A,B). Different dosages of MYCTH_56237 and MYCTH_49824 were selected because these amounts of the enzyme had the best activity to degrade the corn stover (data not shown). Both the MYCTH_56237 and MYCTH_49824 showed synergistic actions by releasing 17.08 and 18.12 mg/mL of reducing sugars, respectively (Figure 5C).

### Homology modeling

In order to understand the structural bases of the high activity of MYCTH_56237 and MYCTH_49824, we next predicted the secondary and tertiary structures. By using PSIPRED, we found both MYCTH_56237 and MYCTH_49824 consist of 14 β-strands and a single α-helix (Supplementary Figure 3).

The tertiary structures of MYCTH_56237 and MYCTH_49824 were generated by the homology module belonging to family GH11 xylanase from *Talaromyces cellulolyticus* (PDB code 3WP3) with resolved three-dimensional structure (Kataoka et al., 2014). The amino acid sequence alignment of TcXylC at the primary structure level displayed a high degree of similarity with MYCTH_56237 and MYCTH_49824, especially in the catalytic sites. ProQ server tools were used to score the constructed models. The LG values of MYCTH_56237 and MYCTH_49824 for the constructed models were 4.28 and 3.981, whereas the MaxSub values were 0.525 and 0.463, respectively. These results corroborated the 3D structural conformations which could be displayed by PyMOL. The overall structures of MYCTH_56237 and MYCTH_49824 resembled a closed right hand (Supplementary Figures 4A,D). The difference was located at the position of the 13th β-strand shown in Supplementary Figures 4B,E. Glu119 and the catalytic nucleophile Glu210 located at β-strands 8 and 13 in MYCTH_56237 and MYCTH_49824, respectively, were found to be at the same position as in template TcXylC. Based on the template, the substrate-binding sites in MYCTH_56237 and MYCTH_49824 were further investigated (Supplementary Figures 4C,F). In subsite −3 for TcXylC, there were three residues: Ile161, Glu162, and Gly163. Six residues: Ser49, Trp52, Tyr112, Pro159, Ser160, and Tyr204 were located at subsite −2. In subsite −1, there were Asp78, Phe79, Thr80, Tyr110, Glu119, Arg155, and Glu210. Seven residues of Asp78, Tyr106, Tyr121, Arg155, Gln169, Trp171, and Glu210 were located at subsite +1. Asn104, Tyr129, and Tyr212 were located at subsite +2 and Tyr129 on subsite +3. These residues have significant roles in enzyme catalysis and substrate binding (Kataoka et al., 2014).

## Discussion

The filamentous fungi have been used as prolific producers of industrial enzymes for almost five decades (Polizeli et al., 2005). *M. thermophila* is considered one of the good sources of gene-encoding extracellular thermophilic xylanases and its complete genome is the first of its kind highlighted for thermophilic filamentous fungus (Berka et al., 2011). This powerful cellulolytic fungus also exhibits advantages such as high expression level and high catalytic activity, along with good stability (Karnaouri et al., 2014). In this study, two novel xylanases, MYCTH_56237 and MYCTH_49824, were identified from *M. thermophila* with significant sequence identity to xylanases classified into the GH11 family.

Many recent studies are centered on the engineering and expression of fungal xylanases using the *P. pastoris* expression system to achieve high yields of recombinant xylanases, such as XynHB from *Bacillus pumilus* (Lu et al., 2016), XynB from *Aspergillus usamii* (Wang et al., 2016), and XynA from *Thermobifida fusca* (Zhao et al., 2015). *P. pastoris*, in contrast to bacterial and other expression systems, is the most preferred expression system for the expression of xylanases owing to the benefit of protein folding, especially post-translational modifications. The present study reports xylanases MYCTH_56237 and MYCTH_49824 with high specific activity from *M. thermophila* for expression in *P. pastoris*.

It is noteworthy that the high specific enzyme activity of recombinant MYCTH_56237 and MYCTH_49824 in comparison to the commercial xylanases from *T. longibrachiatum* and *T. langinosus*. Moreover, the specific xylanase activities of MYCTH_56237 and MYCTH_49824 might be also higher than that of reported other fungal xylanases (Ustinov et al., 2008; Fang et al., 2014; Li et al., 2014; Lu et al., 2016) and the xylanases reported from the *M. thermophila* (van Gool et al., 2012, 2013) albeit with different activity assays, substrates and conditions have been used in different studies. More importantly, the xylanase activities of MYCTH_56237 and MYCTH_49824 can be dramatically elevated through fermentation. The levels of recombinant protein production in fermenter cultures are typically much higher than those in shake-flask cultures. Since the fermentation parameters and cultivation conditions (temperature, pH, DO etc.) are monitored and controlled simultaneously, which affect the cells growth and the production of the recombinant xylanases (Shang et al., 2017).

The glycosylation is known to be the main PTM of secretory proteins in *P. pastoris*, with diverse effects on enzyme properties. The significant effect of glycosylation on the enzyme activity of MYCTH_56237 reflects the occurrence of abundant post-translational modifications (glycosylation) in the process of heterologous expression in *P. pastoris*.

The enzyme characteristics reveal that MYCTH_56237 and MYCTH_49824 exhibit optimal activities at 60°C. This property would give an advantage to MYCTH_56237 and MYCTH_49824 to remain stable up to this temperature. The optimal temperatures of MYCTH_56237 and MYCTH_49824 are also noted to be higher than most other reported fungal xylanases (Liao et al., 2012; Wang et al., 2013; Fang et al., 2014). Furthermore, MYCTH_56237 and MYCTH_49824 adapt to a much wider range of pH (pH 3.0–12.0) than other GH11 xylanases. Often, the fungal xylanases belonging to GH11 are acidic with optimum pH below 5.5 (Paes et al., 2012), such as XynC from *Aspergillus kawachii* and XynA from *Penicillium* sp. 40, which have optimal pH of 2.0 (Kimura et al., 2000) and XYL11B from *Bispora* sp. MEY-1, which has an optimal pH of 2.6 (Luo et al., 2009). However, the optimum pH values of MYCTH_56237 and MYCTH_49824 are close to neutral. Finally, MYCTH_56237 and MYCTH_49824 adapt to various chemical reagents and metal ions. Most of the agents were involved in the activation of the enzyme activity, which was remarkably enhanced in the presence of SDS, tween-20 and EDTA (Gomez et al., 2016). EDTA has no significant inhibiting factor for the xylanase activity, indicating that both the MYCTH_56237 and MYCTH_49824 are independent of metal as a prosthetic group.

The complete hydrolysis of biomass generally requires specific degradation enzymes acting synergistically. The degradation of polysaccharides is regarded as the rate- and cost-limiting step in the production of biofuels. Therefore, the degradation efficiency of xylanases toward different commercial substrates has been demonstrated in different studies (van Gool et al., 2012, 2013). Various pretreatment methods have been intensively used to fulfill the objective of efficient degradation of lignocelluloses into desired end products. The alkaline pretreatment method considered as the most appropriate method which de-lignifies the lignocelluloses and improves the enzymatic digestibility of the residue (Qing et al., 2017). In the present study, the alkaline pretreatment of the corn stover has been applied by sodium hydroxide (NaOH) at 121°C. An effective alkaline pretreatment usually requires a pretreatment temperature higher than 100°C (Qing et al., 2017). Celluclast 1.5 L mainly consists of cellobiohydrolases and endoglucanases (Arantes and Saddler, 2011). Therefore, the performance of MYCTH_56237 and MYCTH_49824 in the process of synergetic saccharification with the commercial Celluclast 1.5 L is certainly a symbolic index to estimate their application value.

When modeling the structure, the MYCTH_56237 and MYCTH_49824 mediated a similar hydrolysis of the glycosidic bond through an acid/base double displacement mechanism that leads to the retention of anomeric configuration according to the TcXylC template (Supplementary Figure 4A). The structure of the main chain shows high similarity with the structures of other GH11 xylanases, including *Acremonium cellulolyticus* (1PVX_A) and *Trichoderma longibrachiatum* (3AKT_B). Sequence alignment of MYCTH_56237 and MYCTH_49824 with described xylanases reveals the high sequence similarities of primary and secondary structures (Supplementary Figure 3). The comparisons among the tertiary structures provide the mechanism of substrate binding.

## Conclusion

In this study, two novel endo-β-1,4-xylanases have been identified from the filamentous thermophilic fungus *M. thermophila*. Many xylanases from different fungal sources have been reported to date. However, MYCTH_56237 and MYCTH_49824 exhibit a high level of specific enzyme activity at the favorable temperature of 60°C with optimal neutral pH. Their rate-limiting performance in saccharification renders them suitable candidates for industrial practices and biofuel production in future.

## Author contributions

AB and JL: designed and performed the experiments; TM and FZ: helped in fermentation; KR: revision of the manuscript; HL and WJ: supervised the present study; AB and HL: prepared the manuscript.

### Conflict of interest statement

The authors declare that the research was conducted in the absence of any commercial or financial relationships that could be construed as a potential conflict of interest.
