# Supplementary material for: Characterization of Two Endo-β-1, 4-Xylanases from Myceliophthora thermophila and Their Saccharification Efficiencies, Synergistic with Commercial Cellulase
Source: Front Microbiol. 2018 Feb 14;9:233. doi: 10.3389/fmicb.2018.00233 (PMC5817056; doi:10.3389/fmicb.2018.00233)
Supplement: Supplementary file 1 [file Image1.PDF]

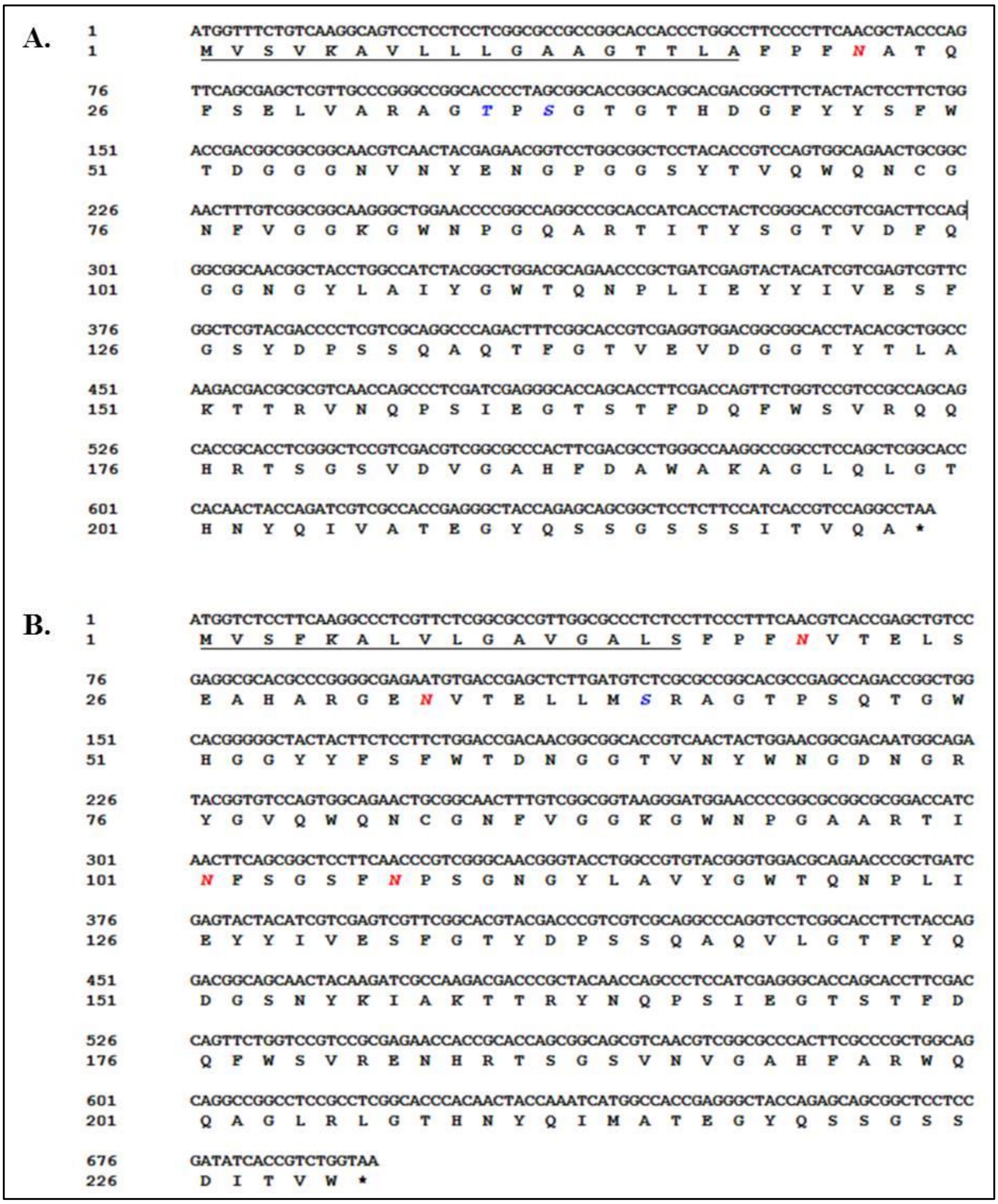

Supplementary Figure 1. Sequence analysis of MYCTH_56237 (**A**) and MYCTH_49824 (**B**). The peptide signal sequence is indicated by underlined. While N- and O- glycosylation sites are highlighted red and blue respectively. The stop codon is marked by asterisk (*).
