# Supplementary material for: Characterization of Two Endo-β-1, 4-Xylanases from Myceliophthora thermophila and Their Saccharification Efficiencies, Synergistic with Commercial Cellulase
Source: Front Microbiol. 2018 Feb 14;9:233. doi: 10.3389/fmicb.2018.00233 (PMC5817056; doi:10.3389/fmicb.2018.00233)
Supplement: Supplementary file 2 [file Image2.PDF]

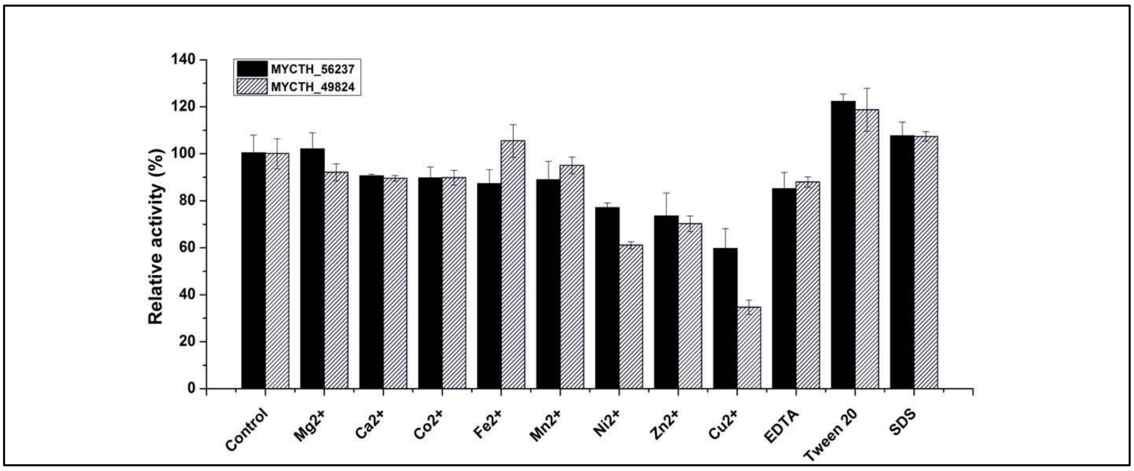

Supplementary Figure 2. Effect of metal cations and additives on xylanase activity of MYCTH_56237 and MYCTH_49824. The effect of various metal cations (at 5 mM) and detergents (0.1% w/v SDS and 0.05% w/v Tween-20) as percent increase or decrease in activity with respect to a control reaction under standard conditions. Error bars represent the standard deviation of two replicated experiments.
