# Supplementary material for: Characterization of Two Endo-β-1, 4-Xylanases from Myceliophthora thermophila and Their Saccharification Efficiencies, Synergistic with Commercial Cellulase
Source: Front Microbiol. 2018 Feb 14;9:233. doi: 10.3389/fmicb.2018.00233 (PMC5817056; doi:10.3389/fmicb.2018.00233)
Supplement: Supplementary file 3 [file Image3.PDF]

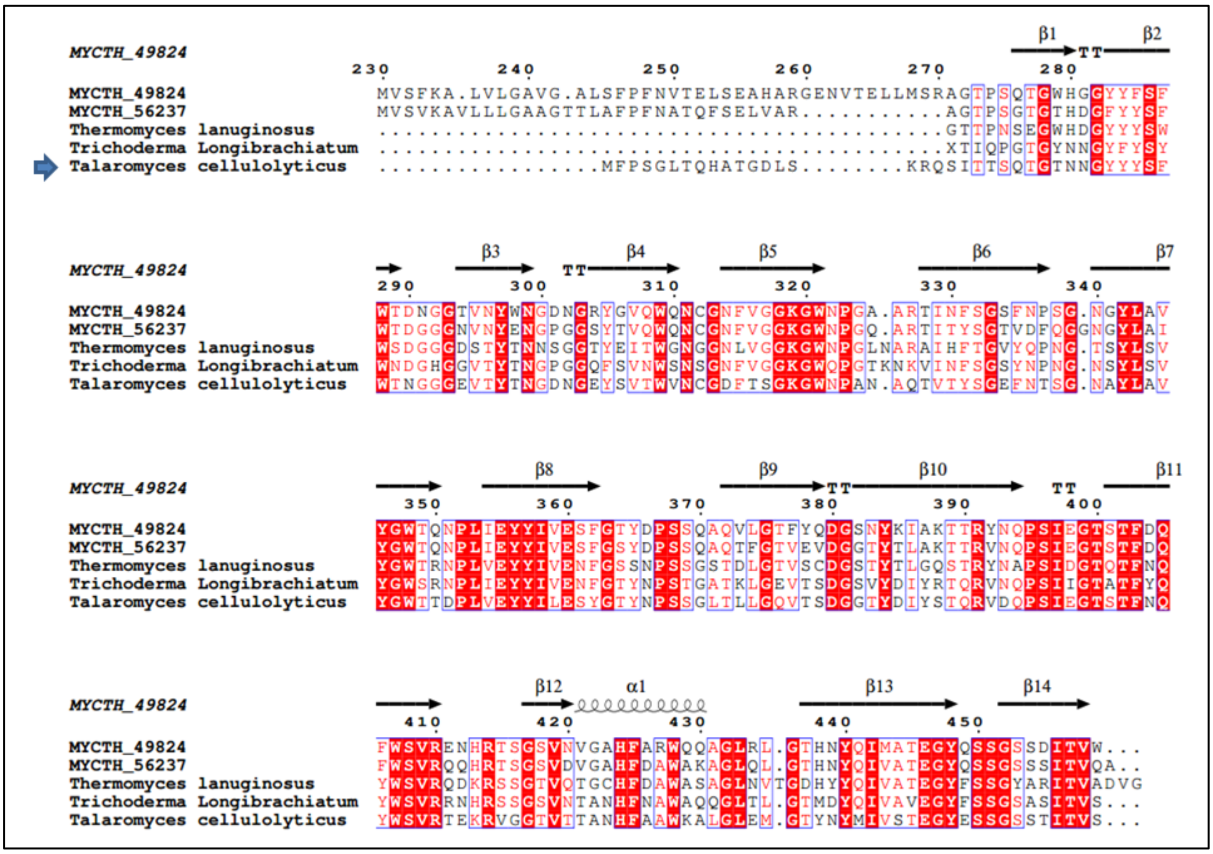

Supplementary Figure 3. Multiple sequence alignment of MYCTH_56237 and MYCTH_49824 and the templates *T. cellulolytic* (PDB code 3WP3), *T. lanuginosus* (PDB code 1YNA), and *T. longibrachiatum* (PDB code 3AKT_B) used for the construction of the 3D model. Arrow indicates the secondary structure elements from the known 3D structure of *T. cellulolytic* (PDB code 3WP3). β-sheets, strict β-turns, and α-helices are denoted β, TT, and α, respectively.
