# Supplementary material for: Characterization of Two Endo-β-1, 4-Xylanases from Myceliophthora thermophila and Their Saccharification Efficiencies, Synergistic with Commercial Cellulase
Source: Front Microbiol. 2018 Feb 14;9:233. doi: 10.3389/fmicb.2018.00233 (PMC5817056; doi:10.3389/fmicb.2018.00233)
Supplement: Supplementary file 4 [file Image4.pdf]

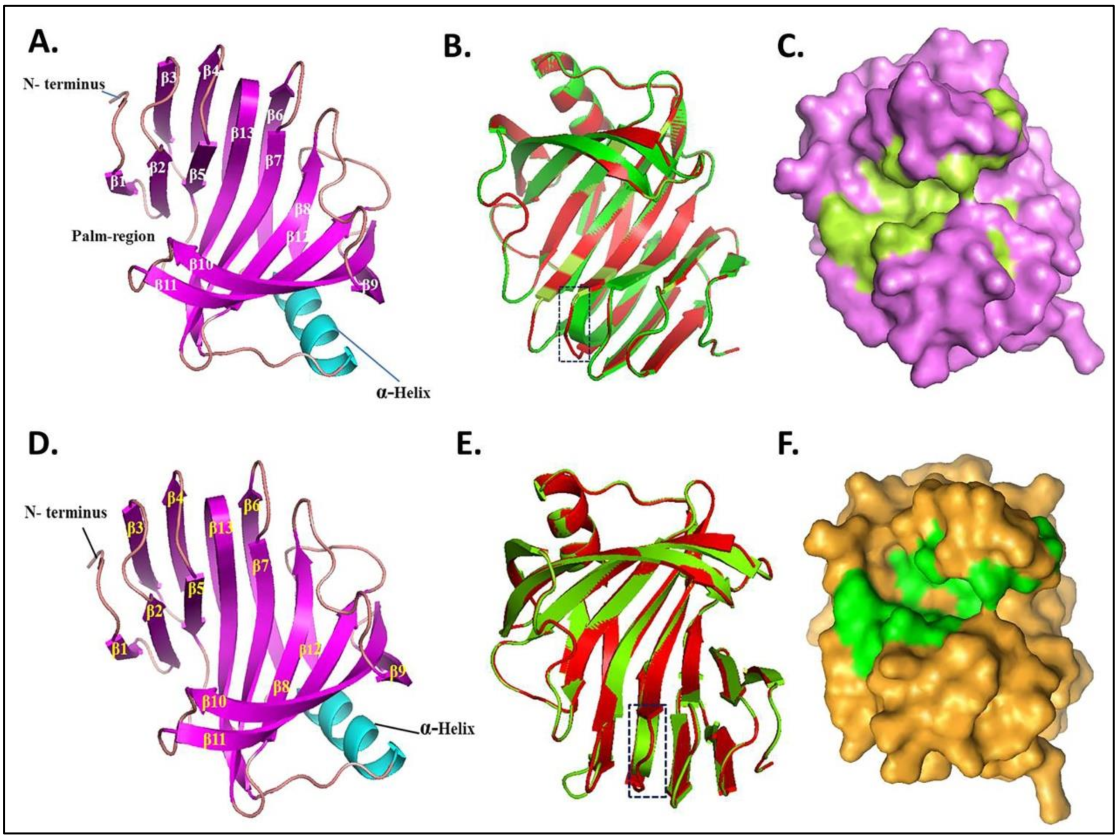

Supplementary Figure 4. Predicted model structures of MYCTH_56237 and MYCTH_49824. **A** and **D:** The structure models of MYCTH_56237 and MYCTH_49824, respectively, using the crystal structure of TcXylC (PDB code 3WP3) as the template. **B** and **E** represent the structure homology, with template TcXylC indicated by red, and MYCTH_56237 and MYCTH_49824 by green. **C** and **F** illustrate the model for the substrate binding sites (indicated by green) of MYCTH_56237 and MYCTH_49824 using the template TcXylC.
