# Supplementary material for: Characterization of Two Endo-β-1, 4-Xylanases from Myceliophthora thermophila and Their Saccharification Efficiencies, Synergistic with Commercial Cellulase
Source: Front Microbiol. 2018 Feb 14;9:233. doi: 10.3389/fmicb.2018.00233 (PMC5817056; doi:10.3389/fmicb.2018.00233)
Supplement: Supplementary file 5 [file Table1.DOCX]

Supplementary Table. Primers used for study

| Host | Primer ID | Primer sequences | Restriction sites |
| --- | --- | --- | --- |
| *p. pastoris* | *MYCTH_56237-***F** | *AATTCTTCCCATTCAACGCTACTCA* | *EcoRI* |
|  | *MYCTH_56237-***R** | *TCTAGAAGCTTGAACAGTGATAGAAG* | *XbaI* |
|  | *MYCTH_49824*-**F** | *GAATTCTCCCATTCAACGTTACTGAA* | *EcoRI* |
|  | *MYCTH_49824*-**R** | *TCTAGACCCCAAACAGTGATGTCAGA* | *XbaI* |
|  | *AOX-* **F** | *GACTGG TTCCAATTGACAAGC* |  |
|  | *AOX-* **R** | *GCAAATGGCATTCTGACATCC* |  |
